# miR-148 Regulates *Mitf* in Melanoma Cells

**DOI:** 10.1371/journal.pone.0011574

**Published:** 2010-07-14

**Authors:** Benedikta S. Haflidadóttir, Kristín Bergsteinsdóttir, Christian Praetorius, Eiríkur Steingrímsson

**Affiliations:** Department of Biochemistry and Molecular Biology, Biomedical Center, Faculty of Medicine, University of Iceland, Reykjavik, Iceland; University of Hong Kong, Hong Kong

## Abstract

The *Microphthalmia associated transcription factor* (*Mitf*) is an important regulator in melanocyte development and has been shown to be involved in melanoma progression. The current model for the role of *Mitf* in melanoma assumes that the total activity of the protein is tightly regulated in order to secure cell proliferation. Previous research has shown that regulation of *Mitf* is complex and involves regulation of expression, splicing, protein stability and post-translational modifications. Here we show that microRNAs (miRNAs) are also involved in regulating *Mitf* in melanoma cells. Sequence analysis revealed conserved binding sites for several miRNAs in the *Mitf* 3′UTR sequence. Furthermore, miR-148 was shown to affect *Mitf* mRNA expression in melanoma cells through a conserved binding site in the 3′UTR sequence of mouse and human *Mitf*. In addition we confirm the previously reported effects of miR-137 on Mitf. Other miRNAs, miR-27a, miR-32 and miR-124 which all have conserved binding sites in the Mitf 3′UTR sequence did not have effects on Mitf. Our data show that miR-148 and miR-137 present an additional level of regulating *Mitf* expression in melanocytes and melanoma cells. Loss of this regulation, either by mutations or by shortening of the 3′UTR sequence, is therefore a likely factor in melanoma formation and/or progression.

## Introduction

The MITF (*Microphthalmia associated transcription factor*) protein is a master regulator of melanocyte development and is important in several other cell types including the retinal pigment epithelium (RPE) of the eye, osteoclasts and mast cells [1]. More recently, MITF was shown to play a role in melanocyte stem cells in the hair follicle [2]. The *Mitf* gene encodes a bHLH-Zip transcription factor of the Myc family [3], [4] which regulates melanocyte differentiation by activating other known melanocyte determinants, including Tyrosinase (Tyr), Tyrosinase-related protein-1 (Tyrp-1) and DCT/Tyrp-2 [5], [6]. In addition to regulating differentiation, MITF is required for melanocyte cell survival [7], [8], proliferation and cell cycle progression [9], [10], [11], [12], [13]. The diverse roles that MITF plays in the development of different cell types suggests that the expression and function of *Mitf* must be highly regulated. In fact, *Mitf* is regulated at multiple levels, including transcription, alternative splicing, post-translational modifications and protein stability. The *Mitf* gene has at least nine different promoters, each with a unique 5′exon which is alternatively spliced to the remaining exons 2 through 9 resulting in several different *Mitf* isoforms [reviewed in 1]. At the protein level, MITF is modified post-transcriptionally and has been shown to be phosphorylated at multiple sites leading to either increase in transcriptional activation or decrease in protein stability [14], [15], [16], [17]. In addition, ubiquitination marks MITF for degradaton [18] and sumoylation has been shown to affect DNA binding specificity [19], [20].

In addition to its essential role in melanocyte development, expression of *Mitf* and its target genes is seen in many melanoma cells [21], [22]. Continued expression of MITF has been shown to be essential for melanoma cell proliferation and survival and, in fact, *MITF* has been proposed to act as a lineage survival oncogene in melanoma [23]. Significantly increased levels of MITF, however, reduce melanoma cell proliferation and tumorigenicity [24], [25]. Consistent with this, there is less MITF expressed in melanoma cells than in normal melanocytes [25]. Therefore, it has been proposed that MITF levels dictate functional outcome such that high levels result in cell cycle arrest and differentiation, intermediate levels promote proliferation and tumorigenesis whereas low levels lead to cell cycle arrest and apoptosis. According to this model, it is extremely important to regulate MITF expression in melanoma cells.

miRNAs have been proven to be involved in many different cell processes including cell-cycle regulation, differentiation, proliferation and apoptosis [reviewed in 26]. miRNAs are thought to regulate about 30% of coding genes in the human genome and to date 677 miRNA genes have been discovered in the human genome and 491 in the mouse (www.microRNA.org) [27]. Misexpression of miRNA genes has been seen in both benign and malignant cancer and miRNA genes can act as either tumor suppressors or oncogenes as is seen for the role of coding genes in cancer progression. Studies have shown that miRNA expression differs between melanoma cell lines [28] and that miRNA expression profiles can be used to classify solid tumors in terms of their differentiation stage and developmental lineage [29]. In an extensive comparative study on miRNA expression in normal melanocyte and melanoma cell lines derived from solid tumors and melanoma metastases, various miRNAs were found to be differentially regulated in melanoma cells compared to normal melanocytes [30]. Many had not been linked to cancer formation before. Other miRNAs had been shown to be oncogenic or to contain tumor suppressive potential in other types of cancer [30].

A few miRNAs have already been linked to *Mitf* expression. Bemis *et al.* (2008) showed that miR-137 downregulates *MITF* in melanoma cells [31]. In addition, it has been shown that miR-182 targets *Mitf* 3′UTR sequence in the retina [32], and recently Segura *et al.* (2009) showed that miR-182 promotes migration and survival of melanoma cells by downregulating *Mitf* expression [33]. Here we study miRNAs with conserved binding sites in the *Mitf* 3′UTR sequence and characterize their effects on *Mitf* using a reporter construct. Effects on the endogenous human *MITF* gene were also determined in melanoma cells. Our results show that miRNAs play an important role in regulating *MITF* mRNA in melanoma through conserved sites in the 3′UTR.

## Materials and Methods

### PCR amplification of the 3′UTR sequence of *Mitf*

A reporter gene construct was generated which contains the mouse *Mitf*-3′UTR sequence downstream of the luciferase reporter gene. The construct was termed mouseMitf-3′UTR-luciferase. To do this, the 3′UTR sequence of the mouse *Mitf* gene (which is contained in a single exon) was amplified from BAC clone rpci-23-9a13t7 using Pfu polymerase and the primers 3UTRSacI-Forw: 5′-gag ctc cga gcc tgc ctt gct ctg-3′ and 3UTRNheI-Rev: 5′-gct agc atg tga aaa acc aaa tgc ttt aat ga-3′. The primers included new restriction sites, SacI at the 5′end and NheI at the 3′end (underlined bases). The restriction sites were used for cloning into the Pis0 vector, a Firefly luciferase vector, modified from the PGL3 Control Vector from Promega by adding restriction sites to the 3′end. The Pis0 vector was purchased from Addgene Inc. Cambridge, MA, USA (Addgene plasmid 12178) [34]. Site directed mutagenesis was used to alter the miRNA target sites using the mouseMitf-3′UTR-luciferase clone as a template.

### MicroRNA molecules

MicroRNAs used in this study were purchased from Ambion, Inc. and are the following: hsa-miR-27a (Product ID:PM10939), hsa-miR-32 (Product ID:PM10124), hsa -miR-101 (Product ID:PM10537), mmu-miR-124a (Product ID:PM10691), mmu-miR-137 (Product ID: PM10513), hsa-miR-148a (Product ID:PM10263), hsa-miR-182. (Product ID:PM11090), Pre-miR-neg#2 (Cat. no. AM17111), Cy3-labeled Pre-miR Negative Control#1 (Cat. no. AM17120).

### MicroRNA inhibitors

anti-microRNA where purchased from Ambion and are the following: anti-miR-137 (Product ID: AM10513) and anti-miR-148 (Product ID: AM10263).

### Cell culture conditions

Human embryonic kidney cells (HEK293) were cultured in DMEM medium with 10% FBS, penicillin-streptomycin (50 U/ml) and 2 mM glutamine. 501mel melanoma cells were cultured in RPMI medium with 10% FBS and penicillin-streptomycin (50 U/ml). MeWo melanoma cells were cultured in DMEM medium with 7% FBS, penicillin-streptomycin (50 U/ml) and 2 mM glutamine. Cells were grown at 37°C with 5% CO_2_.

### Co-transfection

DNA and miRNAs were co-transfected into HEK293 and 501mel cells with Lipofectamine 2000 from Invitrogen (Cat no. 11668-019) in Optimem I + Glutamax-I Reduced Serum Medium from Invitrogen (Cat.no. 51985), according to the manufacturers protocol. Cells were grown in 96 well plates. In each well 10 ng vector DNA, 0.2 ng of Renilla luciferase DNA and an miRNA at either 0.1 pmol, 0.5 pmol or 1 pmol concentration were co-transfected. A negative control was included in all co-transfections. For co-tranfection with the mutated 3′UTR clones we analysed the highest concentration in each case, or 1 pmol miRNA. miRNA inhibitors were transfected at concentration of 1 pmol miRNA. Transfected cells were incubated for 48 hrs. at 37°C with 5% CO_2_ before luciferase expression was measured. Experiments were repeated three times in 6 replicates, except experiments when two different miRNAs were co-transfected simultaneously which was done only once in six replicates.

### Luciferase measurements

The Dual Luciferase Reporter Assay System from Promega was used to measure luciferase expression on a Wallac Victor 2 1420 Multilabel counter.

### Mutagenesis

Locations of miRNA binding sites were determined using the mRNA sequence of mouse *Mitf* 3′UTR and are numbered starting at the first nucleotide after the stop codon. Quikchange Lightning Site-directed Mutagenesis Kit from Stratagene (Cat.no.#10518) was used to mutate the miRNA binding sites in the mouse *Mitf* 3′UTR sequence. Primers were designed using the Primerdesign program from Promega (Table 1). In order to avoid the possibility that the mutated sequence itself forms a microRNA target, the corresponding “new” seed region was used to search for predicted targets of novel small RNAs in TargetscanCustom 4.2. (http://www.targetscan.org/vert_42/seedmatch.html).

**Table 1:** Primers used for mutagenesis.

Primer name	Sequence
miR-124 (548-554)F	5′-ggcaatttcctggtact**cgggccc**agacacagtgccc-3′
miR-124 (548-554)R	5′-gggcactgtgtct**gggcccg**agtaccaggaaattgcc-3′
miR-124 (1639-1646)F	5′-gcataccctttcagaatgaag**ccgggcc**aaaatctcagcagtctc-3′
miR-124 (1639-1646)R	5′- gagactgctgagattttggcccgg cttcattctgaaagggtatgc-3′
miR-137 (2495-2501)F	5′-gctgggagaggcaggc**cccgggc**ttagaggtgacaacataggg-3′
miR-137 (2495-2501)R	5′-ccctatgttgtcacctctaa**gcccggg**gcctgcctctcccagc-3′
miR-137 (2782-2788)F	5′-gccaaaaactgtgc**cccgggc**ctggtcatacccagagcatgatgcag-3′
miR-137 (2782-2788)R	5′-ctgcatcatgctctgggtatgaccag**gcccggg**gcacagtttttggc-3′
miR-137 (2842-2848)F	5′-ggttgtttgtaaacaataa**ccgggcc**agaataaacaaaatgcacagg-3′
miR-137 (2842-2848)R	5′-cctgtgcattttgtttattct**ggcccgg**ttattgtttacaaacaacc-3′
miR-137 (3061-3067)F	5′-gccccccgctgttgggtac**ccgggcc**ctttctgtatggtccgc-3′
miR-137 (3061-3067)R	5′-gcggaccatacagaaag**ggcccgg**gtacccaacagcggggggc-3′
miR-148/152 (1674-1680)F	5′-cagcagtctcttttggaccagca**gccc**ctgaactgtaactaggag-3′
miR-148/152 (1674-1680)R	5′-ctcctagttacagttcag**gggc**tgctggtccaaaagagactgctg-3′
miR-148/152 (2931-2937)F	5′-gttaatatttctgaaaaaaaa**ccgggcc**gggagaagttgatgttg-3′
miR-148/152 (2931-2937)R	5′-caacatcaacttctccc**ggcccgg**ttttttttcagaaatattaac-3′

Mutated bases are bold and underlined.

### Transfection of miRNAs into MeWo cells

Cells were plated at 1.4−2×10^5^ density on 6 well plates. The following day, 20 pmol miRNA or 20 pmol miRNA together with 20 pmol of the anti-miRNA were transfected in triplicate with Lipofectamin 2000 and total RNA isolated for qRT-PCR analysis 48 hrs. post transfection. Subsequently, proteins were extracted and analyzed.

### RNA extraction and cDNA synthesis

Total RNA was extracted from MeWo cells using TRIzol Reagent (Invitrogen), according to the manufacturers protocol [35]. RNA quantity was measured using Nanodrop Spectrophotometer ND-1000 and RNA integrity was determined using Agilent 2100 Bioanalyzer. RNA Integrity number was above 7.6 in all cases. RNA was treated with DNAseI and purified with the RNEasy Minelute kit from Qiagen. Purified RNA (1 µg in 10 µl reaction) was used for cDNA synthesis with Superscript III RT (200 U/µl), and anchored Oligo(dT)_20_ primer according to the manufacturers protocol. The RNA samples were tested for absence of inhibitors using the SPUD assay - no inhibitors were detected [36].

### Quantitative reverse transcription PCR

Human *MITF* mRNA was measured in total RNA samples extracted from MeWo cells, using the human *MITF* FAM-labelled TaqMan Gene Expression Assay (hs01115560) (Applied Biosystems). Samples were measured in triplicate. Two controls were included, one without RT and the other without template. Standard conditions for TaqMan reagents were used. The results were normalized to total RNA (Log_2_50). β-actin was used as a control for the miRNA inhibitor experiment. qRT-PCR results were analysed using the GenEx Std. 4.4.2. software from MultiD analyses AB (Sweden).

### Western blot

Proteins were isolated from the phenol-ethanol supernatant after RNA and DNA had been isolated using the Trizol reagent (Invitrogen), according to the manufacturers protocol [35]. The proteins were separated on a 10% SDS-PAGE polyacrylamide gel. The proteins were electroblotted onto a PVDF transfer membrane (Thermo Scientific). The membrane was immunoblotted overnight at 4°C with primary antibody, (C5 mouse-anti-Mitf monoclonal antibody), in Tris-buffered saline with 5% milk. The C5 mouse-anti-Mitf monoclonal antibody is raised against the N-terminus of MITF. This antibody detects all isoforms of MITF and does not differentiate between them. A horse-radish peroxidase labelled anti-mouse secondary antibody (Jackson) was incubated with the membrane for 1 hr. after washing with TBST. Signals were detected with ECL reagent from Thermo Scientific.

### Endogenous miRNA expression

Total RNA was isolated from MeWo, 501mel and HEK293 cells as before and samples in triplicate were sent to Exiqon for analysis using the miRCURY LNA microRNA PCR system. PCR assays for hsa-miR-137 (MIMAT0000429), hsa-miR-124 (MIMAT0000422), hsa-miR-506 (MIMAT0002878), hsa-miR-148a (MIMAT0000243), hsa-miR-148b (MIMAT0000759) and hsa-miR-152 (MIMAT0000438) were used to measure endogenous miRNA expression.

### Statistical analysis

T-test analysis (for independent groups) was performed on results from the luciferase assays, qRT-PCR results from transfection in MeWo cells and endogenous miRNA expression analysis.

## Results

### The *Mitf* 3′UTR sequence and potential microRNA binding sites

**Figure 1:**
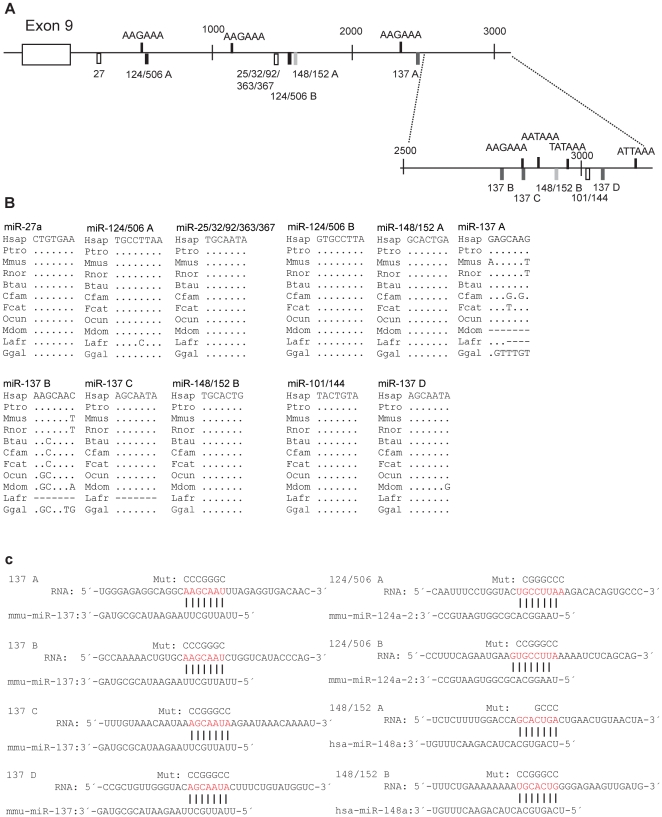
Schematic presentation of the mouse *Mitf* -3′UTR sequence. A. The line indicates the 3′ UTR region of the mouse *Mitf* gene, including the coding region of exon 9. Potential binding sites for miR-27, miR-124/506, miR-25/32/92/363/367, miR-148/152, miR-137 and miR-101/144 in the *mMitf* 3′UTR sequence are indicated below the line and potential PAS sites above. Positions are based on the mouse *Mitf* 3′UTR sequence and are numbered starting at the first nucleotide after the stop codon. Black bars: miR-124/506 binding sites, dark grey bars: miR-137 binding sites, light grey bars: miR-148/152 binding sites, white bars: miR-27, miR-25/32/92/363/367 and miR-101/144. B. The conservation of potential miRNA binding sites in the *Mitf* 3′UTR sequence. Dots represent a conserved base, short lines represent absence of a sequence at this location. C. Mutations made in the *Mitf* 3′UTR sequence. The wild type mouse *Mitf* mRNA sequence is shown with potential binding sites indicated in red. The mature miRNA sequence and potential binding between the miRNA seed region to the 3′UTR sequence are shown and mutated bases are indicated above the mRNA sequence.

The mouse and human *Mitf* 3′UTR sequences are unusually long or over 3 kb in length. This is considerably larger than the average mouse and human 3′UTR sequences which are 559 and 826 nucleotides, respectively [37]. The overall conservation of the *Mitf* 3′UTR sequence in 11 vertebrate species is 36% [38] which is very high for a non-coding region. This suggests a functional relevance of the 3′UTR sequence of *Mitf*. Using the TargetScan program (www.targetscan.org), we identified several miRNA binding sites located in the most conserved areas of the 3′UTR region. We tested the effects of microRNAs which have conserved binding sites in the 3′UTR region of *Mitf*, including miR-27a (located at 229–235 in the mouse *Mitf* 3′UTR sequence), miR-25/32/92/363/367 (1491–1497), miR-101/144 (3023–3029), miR-124/506 (1639–1646) and miR-148/152 (1674–1680 and 2931–2937) (Fig. 1A and 1B). miR-124/506 also has a less conserved binding site at 548–554, and was therefore also tested in our study. In addition, the potential binding sites for miR-137 were tested as the mouse and human *Mitf* 3′UTR sequences both contain two well conserved miR-137 binding sites located in close proximity to each other, 137C (2842–2848) and 137D (3061–3067). Two additional less conserved miR-137 binding sites were found, one only in the mouse 3′UTR, namely 137A (2495–2501) and one conserved in the mouse and rat 3′UTR sequences, 137B (2782–2788). It has been shown that single miRNAs with multiple binding sites in a 3′UTR sequence have increased effects as compared to miRNAs with only a single binding site [39], [40]. This is also true when the binding sites are located in close proximity to each other [41]. In some cases, several different miRNA molecules can potentially bind to the same binding site. In these cases only one representative miRNA molecule was chosen to test the functionality of that specific binding site. If the representative miRNA was able to downregulate via the binding site, it indicates that the other potential miRNAs may also be able to regulate *Mitf* expression. miR-182 has been shown previously to target the 3′UTR of *Mitf* and was used as a positive control [32].

### miR-148/152 affects *mMitf* RNA in melanoma cells

**Figure 2:**
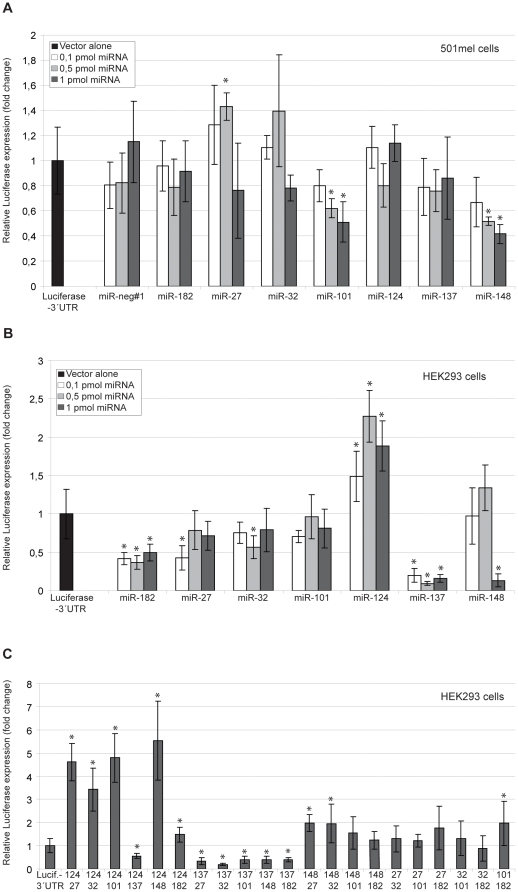
Effects of microRNAs on the *Mitf*-3′UTR-luciferase reporter. A. Effects of miRNAs on the mouseMitf-3′UTR-luciferase reporter in 501melanoma cells. Three concentrations of miRNAs were tested: 0.1 pmol, 0.5 pmol and 1 pmol. Results in all panels are presented as mean values ±SD. Difference between the mouseMitf- 3′UTR-luciferase vector with and without co-transfected miRNA was considered statistically significant when p<0.05, shown with an asterisk (T-test, 95% confidence level). P-values are presented in Table S1. B. Effects of miRNAs on the mouseMitf- 3′UTR-luciferase vector in HEK293 cells. Three concentrations of miRNAs were tested: 0.1 pmol, 0.5 pmol and 1 pmol. C. Effects of miRNAs when transfected in combination with another miRNA on the reporter. Concentration of each miRNA is 0.5 pmol.

To test the functionality of the miRNA binding sites, the mouse *Mitf* 3′UTR sequence was cloned downstream of a luciferase reporter gene. This vector was then co-transfected into cells together with single miRNA molecules and effects of the specific miRNAs on expression determined by measuring luciferase activity. In 501mel cells, miR-148 had significant effects on expression of the mouseMitf-3′UTR-luciferase reporter (Fig. 2A). Co-transfection with miR-101 resulted in some downregulation of the reporter whereas other miRNAs tested did not show statistically significant effects compared to the mouseMitf-3′UTR-luciferase vector alone. When determining how effective each miRNA was in our assay, the expression from the vector when co-transfected with the miRNA was compared to the expression from the vector alone.

### miR-137 and miR-124/506 affect m*Mitf* RNA in HEK293 cells

The luciferase assay experiments were also performed in human embryonic kidney cells (HEK293), which do not express *Mitf* at significant levels. The effects of the miRNAs in HEK293 cells are different from the effects seen in 501mel melanoma cells. In HEK293 cells the microRNA miR-137 negatively affected the mouseMitf-3′UTR-luciferase reporter construct, compared to the reporter construct alone (Fig. 2B). miR-148 affected the reporter only when transfected at the highest concentration (1pmol) (Fig. 2B). miR-124, however resulted in significant increase in reporter gene expression. The other miRNAs tested, miR-27, miR-32 and miR-101 did not show significant effects on luciferase expression in this assay (Fig. 2B). This is surprising as the binding sites for these three miRNAs are very well conserved. All *Mitf* 3′UTR sequences in 11 vertebrate species analysed contain the miR-27, miR-25/32/92/363/367 and the miR-101/144 binding sites (Fig. 1B). In addition, seven species contain a conserved second miR-101/144 binding site. Despite this conservation, these sites do not seem to be regulated by miRNAs in our assays.

The different results in melanoma cells as compared to HEK293 cells might be explained by different levels of endogenous miRNA expression in these cell types. If one of the miRNAs tested has high endogenous expression in one of the cell types it would affect the expression of the reporter alone. Adding more of the miRNA would presumably not make a major difference. Thus, the effects of the transfected miRNA would be masked by the endogenous expression of the miRNA.

Many different miRNAs can bind simultaneously to a specific 3′UTR sequence and affect expression. Thus, we tested if transfecting two different miRNAs simultaneously would reveal additive effects. The results are compared to expression by the mouseMitf-3′UTR-luciferase reporter alone. When miR-137 was combined with other miRNAs, the effects were always equal to the effects observed with miR-137 alone. Also, when miR-124 was combined with other miRNAs, the effects were always equal to the effects observed with miR-124 alone. When miR-124 and miR-137 were combined, the same effect was observed as when miR-137 was transfected alone (Fig. 2C). Other combinations of two miRNAs did not show increased effects. Thus, we detect no cooperation among the miRNA molecules with respect to effects on *Mitf*.

### Roles of individual miRNA target sites revealed through mutagenesis

To confirm that the miRNAs which show effects on the mouseMitf-3′UTR-luciferase reporter are in fact operating through the predicted potential binding sites, the miRNA binding sites were mutated in the mouseMitf-3′UTR-luciferase vector and the vector then co-transfected into cells together with the specific miRNAs, as before. miRNAs bind to their 3′UTR binding site through a seed region located at position 2–7 or 2–8 in the mature miRNA sequence [27], [42]. We mutated all 7 bases in the 3′UTR binding site matching the seed region of the mature miRNAs in order to abolish completely the binding of the miRNA. In the case of one of the miR-148 binding site, 148/152A, only 4 bases were mutated as a clone with the fully mutated binding site was not generated successfully. The mutations are shown in Fig. 1C and the primers used for mutagenesis in Table 1.

#### Role of the miR-148/152 target sites

**Figure 3:**
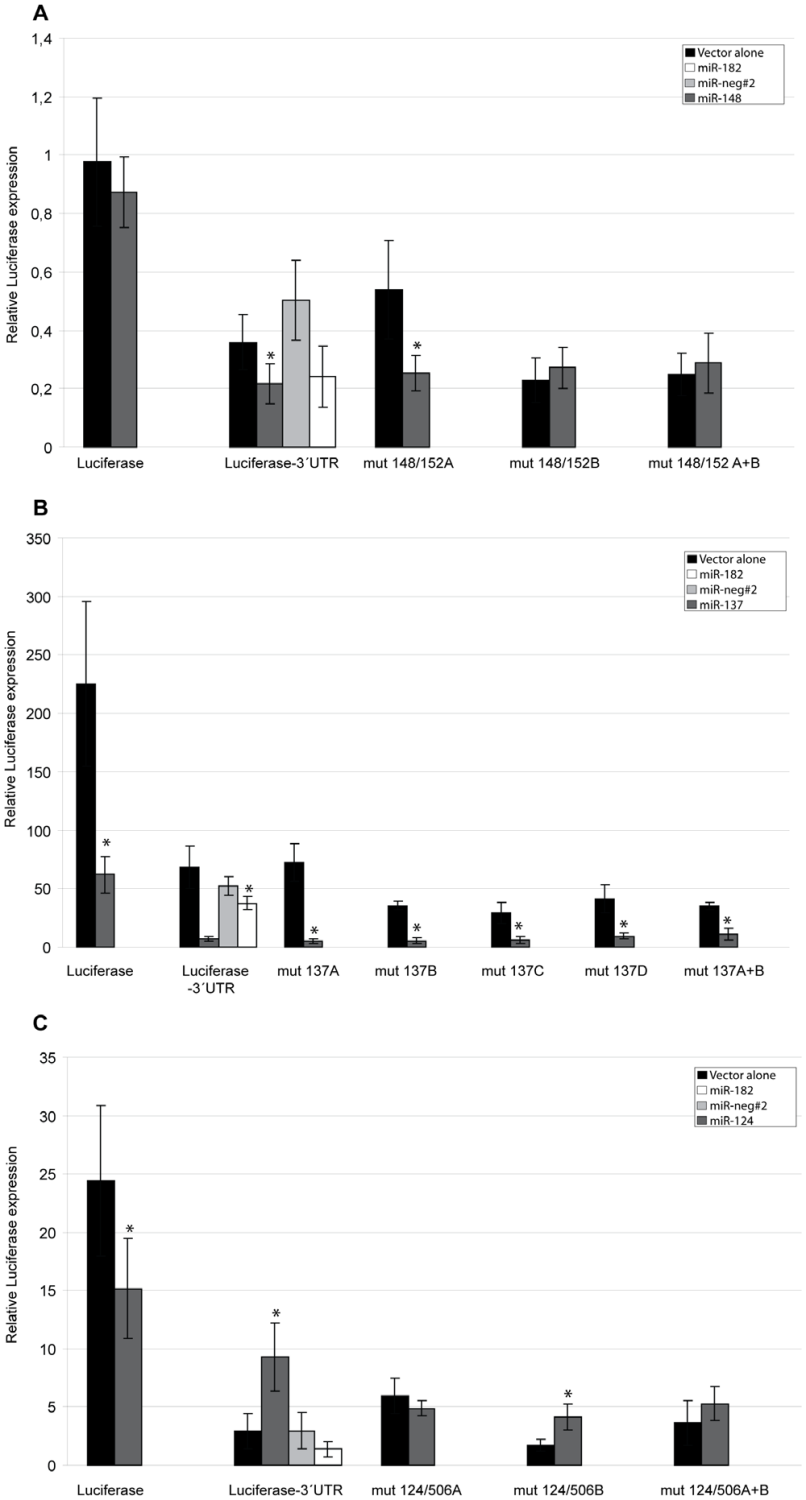
Mutating miRNA binding sites affects the *Mitf* mRNA. A. Effects of miR-148 on the mouseMitf-3′UTR-luciferase reporter when the potential binding sites are mutated. Results in all panels are presented as mean values ±SD. Difference between the appropriate vector (labeled on the X-axis) with and without co-transfected miRNA was considered statistically significant when p<0.05, shown with an asterisk (T-test, 95% confidence level). P-values are presented in Table S1. B. Effects of miR-137 on the mouseMitf-3′UTR-luciferase reporter when the potential binding sites are mutated. C. Effects of miR-124 on the mouseMitf-3′UTR-luciferase reporter when the potential binding sites are mutated.

There are two target sites for miR-148/152 in the mouse *Mitf* 3′UTR sequence. Each of the two sites were mutated, 148/152A located at 1674–1680, and 148/152B located at 2931–2937 (Fig 1A). In these experiments, we define the expression level of the vector alone as 100%. When determining how effective each mutation was in our assays, expression of luciferase from the mutated vector alone was compared to expression from the mutated vector when co-transfected with the appropriate miRNA. When both binding sites were functional miR-148 downregulated reporter gene expression to 50–60% compared to expression from the vector alone in 501mel cells (p = 0.0140) (Fig. 3A). When the 148/152A binding site was mutated, miR-148 downregulated reporter gene expression to 47% (p = 0.0049). However, when the 148/152B binding site was mutated, miR-148 was no longer able to downregulate reporter gene expression (p = 0.3929). When both binding sites were mutated, reporter gene expression was the same as when 148/152B was mutated (118%, p = 0.4918). These results indicate that miR-148 is able to downregulate *Mitf* expression by binding to the 148/152B binding site.

#### Role of the miR-137 target sites

The four target sites for miR-137 in the Mitf 3′UTR sequence, 137A (2495–2501), 137B (2782–2788), 137C (2842–2848) and 137D (3061–3067) were mutated seperatly. In addition, a double mutant was created where the most conserved sites 137C and 137D were changed simultaneously, a triple mutant with 137B, 137C and 137D mutated and a mutant where all four potential binding sites were changed (Fig. 1C). The mutated mouseMitf-3′UTR-luciferase constructs were co-transfected together with the miRNAs in HEK293 cells. When all the miR-137 binding sites are functional, miR-137 reduced expression from the luciferase reporter to only 10% (p = 0.0015) compared to the vector alone (Fig. 3B).

When miR-137 was co-transfected with a reporter construct containing the mutant site 137A, expression from the luciferase reporter was 7% compared to the vector alone (p<0.0001). This is similar reduction as was seen with the wild type construct, suggesting that this site has no role in the response to miR-137 (Fig. 3B). Mutating the 137B site resulted in 16% expression (p<0.0001) which is a little less reduction than was seen when all the potential binding sites were functional. When the more conserved site 137C was mutated, expression from the mouseMitf-3′UTR-luciferase reporter was reduced to 19% (p = 0.0001) (Fig. 3B), indicating a functional role of miR-137C. Mutating the other well conserved binding site 137D resulted in reduction to 23% expression (p = 0.0001) of the mutated mouseMitf-3′UTR-luciferase reporter (Fig. 3B), again indicating a functional target site. When the two best conserved binding sites (137C and 137D) were simultaneously mutated, reporter expression was 32% (p = 0.0001) compared to expression from the mutated vector alone, suggesting that sites C and D play a major role in the response to miR-137. Surprisingly, when miR-137 was co-transfected with a reporter construct lacking the 3′UTR sequence of *Mitf*, miR-137 downregulated luciferase expression to 27% (Fig. 3B). This suggests that the luciferase vector has binding sites for miR-137. Indeed, our search for miR-137 binding sites in the luciferase gene itself resulted in 3 matching sequences. There are reports showing that binding sites within the coding regions of genes can result in effects on gene expression [43]. It is also possible that the miR-137 is affecting other components (genes/transcripts) that are involved in the transcription of the luciferase gene. Thus, when sites 137C and 137D were mutated simultaneously, the expression of the reporter was reduced to 32% which is very similar to the level of expression when all miR-137 binding sites are funcional (27%), suggesting that all the effects of miR-137 on the mouseMitf-3′UTR-luciferase are mediated through these two sites. When three or all four miR-137 binding sites were mutated simultaneously, the mutated vector alone was unable to express the luciferase gene (data not shown). One possible explanation for this might be that the structure of the 3′UTR is altered when 21 or 28 conserved bases are mutated, leading to reduced stability or effects on translation of the message. miR-137 has previously been shown to target *Mitf* by acting through the best conserved miR-137 target sites [31]. However, in that study, only a portion of the Mitf 3′UTR sequence was used in the analysis [31]. In our analysis, however, the entire 3′UTR sequence from mouse *Mitf* (over 3 kb) was used to analyse the role of the binding sites. This more closely resembles the endogenous situation. Our results therefore confirm the role of miR-137 in regulating *Mitf* expression.

#### Role of the miR-124/506 target sites

There are two binding sites for miR-124/506 in the mouse *Mitf* 3′UTR sequence. Site 124/506A is located at 548-554 and site 124/506B at 1639-1646 in the mMitf 3′UTR sequence. When both binding sites are functional, miR-124 positively affects luciferase expression up to 3 fold compared to expression from the vector alone in HEK293 cells (p = 0.0086) (Fig. 3C). We mutated each target site alone and also made a double mutation changing both sites. When the 124/506A binding site was mutated, miR-124 no longer affected expression of the reporter gene. The expression was only 81% compared to 315% in the vector alone (P = 0.1376). When the 124/506B binding site was mutated, miR-124 still upregulated lucifease expression to 240% (p = 0.0030). When both binding sites were mutated, miR-124 did not result in increased expression of the luciferase reporter, at least not as effectively as when both potential binding sites were functional (146% expression, p = 0.1864). These results suggest that miR-124 leads to positive effects on the *Mitf* mRNA by binding to the 124/506A binding site. At present, it is not clear how miR-124 mediates this upregulation. The effects of miR-506, that binds to the same target site, were not tested.

### miR-148 and miR-137 affect expression of the endogenous *MITF*

**Figure 4:**
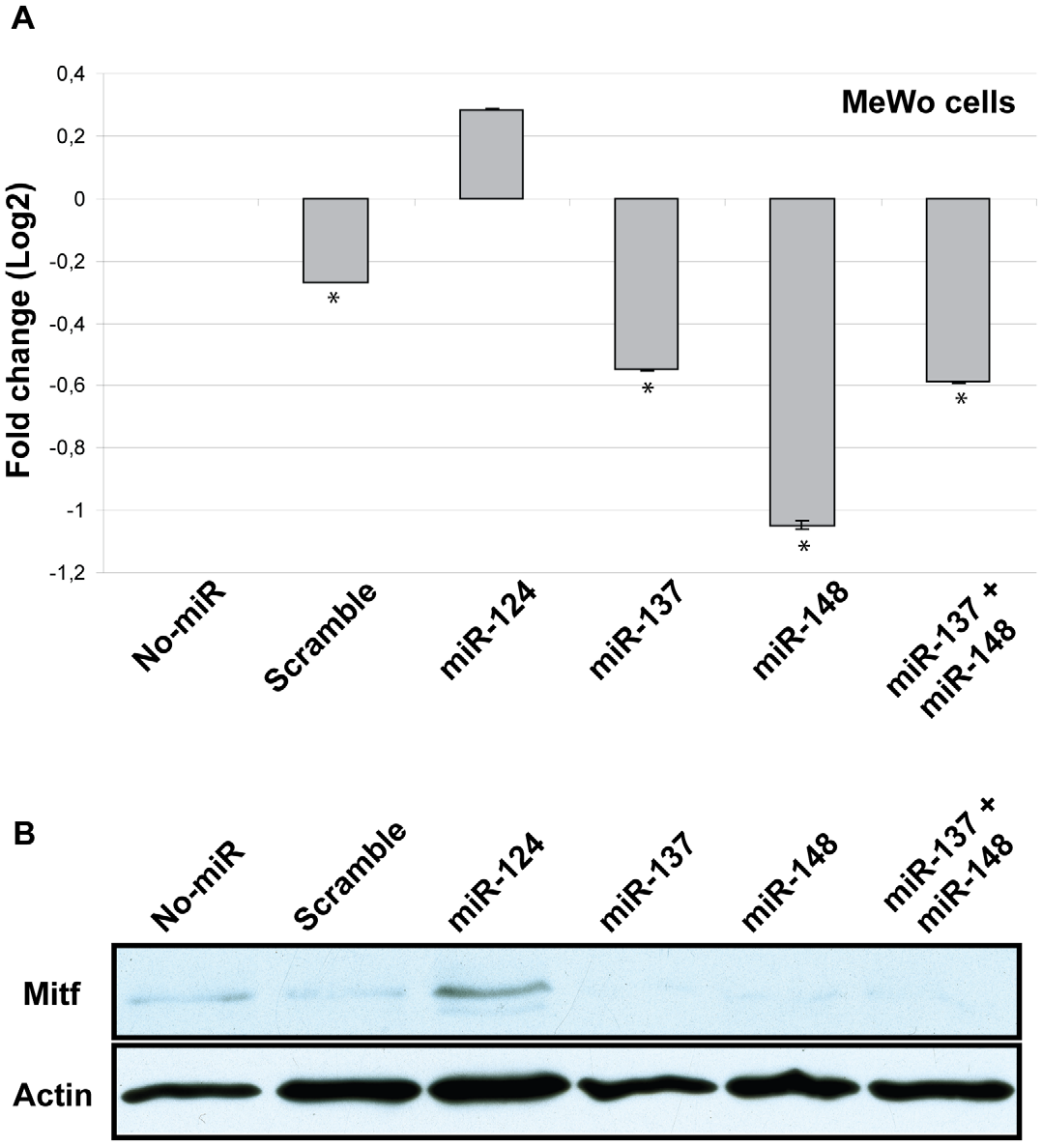
Effects of miRNAs on endogenous *MITF.* A. Expression of human *MITF* mRNA in MeWo cells transfected with miR-124, miR-137, miR-148 or miR-137 and miR-148 combined. The figure shows average expression levels of three replicates for each sample relative to untreated cells (No-miR). Negative control is a scramble miRNA sequence (scramble). Statistically significant difference between untreated cells and treated cells are shown with an asterisk (T-test, 95% confidence level). P-values are: Scramble  = 0.0396; miR-124 = 0.2228; miR-137 = 0.0069; miR-148 = 0.0051; miR-137+148 = 0.0051. B. Western blot analysis on MITF protein levels in MeWo cells, when transfected with miR-124, miR-137, miR-148 or miR-137 and miR-148 combined.

In order to test if these miRNAs affect the level of endogenous *MITF* mRNA in melanoma cells, we used qRT-PCR to determine *MITF* mRNA expression after transfecting MeWo melanoma cells with the miRNAs miR-124, miR-137 and miR-148. MeWo cells express less *MITF* than the 501mel cells used previously. Therefore, the difference in expression upon treatment was more easily detected with the amount of miRNA used. The results, normalized to total RNA, are shown in Fig. 4A and confirm our previous findings. When miR-137 was transfected into MeWo cells, the level of *MITF* mRNA was reduced to 68% (Cp value 22.3, p = 0.0069) compared to 100% in untreated cells (No-miR, Cp value 21.7). Similarily, when miR-148 was transfected into these cells, *MITF* mRNA level was reduced to 48% (Cp value 22.8, p = 0.0051) compared to untreated cells. Simultaneous transfection with miR-137 and miR-148 did not result in further effects on *MITF* expression as it reduced expression to 67% (Cp value 22.3, p = 0.0051), which is similar to the reduction seen with miR-137 alone (Fig. 4A). Thus, these miRNAs do not seem to work synergistically in downregulating *MITF*.

Similar to what we observed using the luciferase-reporter assay, miR-124 was able to upregulate *MITF* expression in the MeWo cells. The expression was increased to 122% (Cp value 21.5) compared to untreated cells; however, this difference is not statistically significant (p = 0.2228). The same experiment was performed once in 501mel cells, the only signifant downregulation was by miR-137 when compared to transfection with a scramble miRNA (data not shown). These results show that the miR-137 and miR-148 have the same effects on expression of the endogenous *MITF* gene as seen using the luciferase reporter assay. The effects of miRNAs on MITF protein level were also tested in MeWo cells and confirmed our previous findings (Fig. 4B). When the cells were transfected with either miR-137, miR-148 or simultaneously with both miR-137 and miR-148, MITF protein levels were reduced. Also, when miR-124 was transfected, higher MITF protein levels were observed (Fig. 4B).

**Figure 5:**
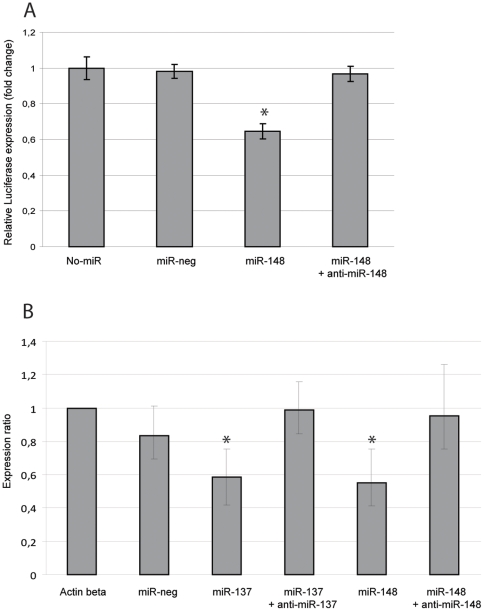
Inhibiting the miRNAs blocks their effect. A. Co-transfection of anti-miR-148 and miR-148 simultaneously with the Mitf-3′UTR-luciferase vector inhibits the effect of miR-148. Results are presented as mean values ±SD. Expression levels were compared to a sample where no microRNAs were added (No-miR) and were considered statistically significant when p<0.05, shown with an asterisk (T-test, 95% confidence level). The p-values are; miR-neg  = 0.14 miR-148 = 0.007, miR-148+anti-miR-148 = 0.119. B. Transfecting miRNAs and anti-miRNAs blocked the effect of the miRNAs on endogenous human *MITF* mRNA levels in MeWo cells. P-values when compared to the no-miR sample are: Neg control  = 0.064, miR-137 = 0.006, miR-137 + anti-miR-137 = 0.854, miR-148 = 0.004, miR-148 + anti-miR-148 = 0.632.

In order to test the specificity of the miRNAs, we used specific anti-miRNAs to inhibit the effects of the miRNAs used in our experiments. Consistent with previous results, cells simultaneously transfected with the luciferase vector construct, the miR-148 and the anti-miR-148 showed that the anti-miR148 molecule effectively inhibited the effects of miR-148 (Fig. 5A). Similarly, the effects of miR-137 and miR-148 on endogenous *MITF* mRNAs are inhibited when MeWo cells were co-transfected with anti-miRNAs (Fig. 5B). These results further verifies that the effects on *Mitf* mRNA are specific to these microRNAs.

### Endogenous miRNA expression

The expression of miRNAs 137, 124, 506, 148a, 148b and 152 was determined in the HEK293, 501mel and MeWo cell lines. The expression of the miRNAs was measured using the MiRcury LNA Universal RT microRNA PCR method. An RNA pool from 20 different tissues was used as a positive control and the results were normalized to the expression of miR-16 and miR-103. Cp values (crossing point) are shown in Table 2.

**Table 2:** Cp values for determining endogenous miRNA expression.

miRNA	HEK293	501mel	MeWo	Positive contr.
**miR-137**	ND (1/9)	ND (0/9)	34.69 (7/9)	37.39 (1/3)
**miR-124**	34.37 (9/9)	34.73 (5/9)	35.09 (2/9)	32.68 (3/3)
**miR-506**	35.19 (2/9)	34.73 (3/9)	32.87 (9/9)	ND (0/9)
**miR-148a**	27.90 (9/9)	28.29 (9/9)	28.82 (9/9)	33.12 (3/3)
**miR-148b**	28.37 (9/9)	28.65 (9/9)	29.37 (9/9)	35.19 (1/3)
**miR-152**	34.08 (9/9)	35.12 (9/9)	34.26 (9/9)	35.05 (2/3)
**miR-16 contr.**	26.19 (9/9)	25.72 (9/9)	25.92 (9/9)	31.83 (3/3)
**miR-103 contr.**	27.07 (9/9)	26.76 (9/9)	27.81 (9/9)	33.09 (3/3)

Cp values for average normalised values are shown for each miRNA tested in HEK293, 501mel and MeWo cells as well as the positive control. Each assay was preformed in triplicate for three RNA samples from each cell type. The number of samples that each miRNA was detected in, is shown in parenthesis. ND  =  not detected.

#### miR-148 and miR-152

**Figure 6:**
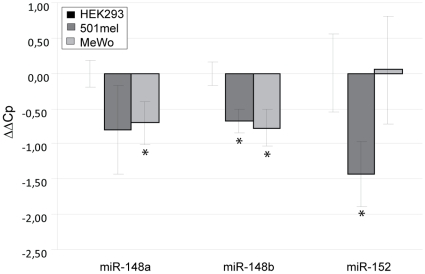
Endogenous miRNA expression. Expression of endogenous miRNAs miR-148a, miR-148b and miR-152 in HEK293, 501mel and MeWo cells. The figure shows average expression levels of three replicates for each sample relative to HEK293 cells. Expression was normalized to miR-16 and miR-103. Statistically significant levels of expression in MeWo and 501mel compared to HEK293 (p<0.05) are shown with an asterisk. P-values are: miR-148a expression in 501mel  = 0.0940 and in MeWo  = 0.0148. miR-148b expression in 501mel  = 0.0023 and in MeWo  = 0.0065. miR-152 expression in 501mel  = 0.0054 and in MeWo =  0.9316.

The microRNAs miR-148a and miR-148b have similar expression levels in all the cell types tested. Expression in the melanoma cell lines 501mel and MeWo was around 1.5 fold less than expression in HEK293 cells (Fig. 6). Higher endogenous expression in HEK293 cells might explain why transfected miR-148 had no effect on reporter gene expression in HEK293 cells, except at the highest concentration. miR-148 affected reporter gene expression more significantly in 501mel cells. Expression of miR-152 in 501mel was 2.7 fold lower than in HEK293 cells and was much lower then the expression of miR-148a and miR-148b in all cell types (see Table 2). Low expression of miR-148a, 148b and 152 in the melanoma cell lines is therefore consistent with the relatively high level of *MITF* expression in these cells.

#### miR-137

Endogenous miR-137 was only detected in MeWo cells, not in HEK293, 501mel or the positive control. Expression in the MeWo cells, although at rather low levels (average Cp value 34.7 compared to the controls (miR-16 and 103) at 25.9 and 27.8 respectively), indicates possible involvement in regulating the endogenous levels of *MITF* in this cell type. *MITF* is expressed in MeWo cells but at a lower level than in 501mel cells which is consistent with the expression of miR-137 in MeWo cells and its absence in 501mel cells. miR-137 affected reporter gene expression in HEK293 cells, which is in line with the absence of endogenous miR-137 in this cell type. It is unclear why transfected miR-137 did not affect reporter gene expression in 501mel cells where endogenous miR-137 was not detected. The absence of miR-137 in 501mel cells might be explained by mutations in the miR-137 gene itself as has been shown to be the case in other melanoma cells [31], thus allowing high levels of *MITF* expression.

#### miR-124 and miR-506

The level of miR-124 expression was considered to be too low to be detected in all cell types. We therefore can not definitely conclude anything about the expression of miR-124 in our samples. miR-506 was detected in 501mel and MeWo cells. The expression level was higher in MeWo cells (Cp value 32.9) compared to 501mel cells (Cp value 34.7). Lower expression in 501mel might be a factor allowing a high level of *MITF* expression in this melanoma cell type.

## Discussion

We have tested the role of six different miRNAs with conserved potential binding sites in the *Mitf* 3′UTR sequence for their ability to affect *Mitf* mRNA expression. We show that miR-137 and miR-148 negatively affect *Mitf* mRNA in melanoma cells through conserved binding sites in the 3′UTR sequence. Other miRNAs tested, miR-27 and miR-25/32/92/363/367 have highly conserved binding sites but did not have an effect on reporter gene expression in HEK293 or 501mel cells. The microRNA miR-101 has a highly conserved binding site but did not affect reporter gene expression in HEK293 cells. However in 501mel cells, miR-101 downregulated reporter gene expression at the two highest concentrations used suggesting that it may play a role in melanocytes or melanoma cells.

The roles of miR-137 and miR-148 were analysed further by mutating the binding sites in the mouseMitf-3′UTR-luciferase reporter construct. The results of this analysis suggest direct effects on the *Mitf* mRNA. When the 148/152B binding sites were mutated, the effects of miR-148 were eliminated. When the most conserved binding sites for miR-137 (137C and D) were mutated, the effects of miR-137 were eliminated. None of the miR-137-mutations restore luciferase expression to the level observed with the negative control. This might indicate that all the miR-137 binding sites contribute to the regulation of Mitf. However, it should be noted that miR-137 can target the luciferase construct lacking the 3′UTR sequence such that in the presence of miR-137, luciferase expression cannot reach the same level of expression as is seen with the negative control (miR-neg#2).

The effects of the miRNAs were further confirmed by transfecting the microRNAs into MeWo melanoma cells and then measuring the endogenous *MITF* mRNA and protein levels. miR-137 and miR-148 both led to reduced levels of *MITF* mRNA and protein. Simultaneous transfection with anti-miR-148 blocked the effect of miR-148 in both the luciferase reporter assays and on the endogenous *MITF* mRNA. Transfection with anti-miR-137 similarly blocked the effects of miR-137 on the endogenous *MITF* mRNA (Fig. 5B). miR-137 did not significantly affect the luciferase reporter in melanoma cells. However, simultaneous transfection of anti-miR-137 and miR-137 with the Mitf-3′UTR luciferase in MeWo cells blocked the effect of miR-137 (data not shown).

Combining both miR-137 and miR-148 did not result in added effects on endogenous *MITF* mRNA levels. Possible explanations for this might be that the maximum effects are reached with one of the miRNAs and adding another does not increase the effects. Another explanation for the lack of additive effects is that the miRNAs might not be able to bind simultaneously to the 3′UTR sequences. The 137C, 137D and 148/152B binding sites are all located in close proximity to each other in the Mitf-3′UTR sequence (Fig. 1A). Accessibility might be limited by one of the miRNAs. A third possibility is that specific binding proteins block the other sites when one is occupied. Specific RNA binding proteins have been show to block the binding of miRNAs to the *MITF* mRNA [44]. The fact that miR-137 has an effect in MeWo cells (q-RT-PCR) and not in 501mel cells might be due to the fact that *MITF* mRNA levels are higher in 501mel cells than in MeWo cells. Thus, more miR-137 might be needed in 501mel cells then in MeWo cells. The effects of miR-137 and miR-148 when transfected seperatly, are blocked when the cells are transfected with miR-137 and miR-148 inhibitors. miR-124 resulted in increased luciferase expression as seen using the reporter gene construct in HEK293 cells. Furthermore, we observed upregulation of the endogenous *MITF* in MeWo cells at both mRNA and protein levels; the upregulation of *MITF* mRNA was not statistically significant. The effects of miR-124 might be an artifact of the reporter system. The effects on endogenous *MITF* could not be measured in HEK293 cells as there is no endogenous *MITF* expression in those cells.

As mentioned earlier, miR-137 has previously been shown to target *MITF* in melanoma cells [31]. Interestingly, searching for miR-137 binding sites within the *Mitf* 3′UTR sequence of 11 vertebrate species revealed that all species have at least two miR-137 binding sites and some species have three or four binding sites. All species contain either 137C or 137D and nine contain them both (data not shown). miR-137 expression has been shown to be affected in cancer. It is silenced by DNA methylation in colorectal cancer [45] and oral carcinogenesis [46], suggesting tumor suppressor abilities. In addition, both miR-137 and miR-124 induced differentiation of adult mouse neural stem cells and were able to induce cell cycle arrest of glioblastoma multiforme cells [47]. Upregulation of miR-137 in lymph node metastasis in colon cancer has also been observed [48].

The 3′UTR sequences of *Mitf* in 11 vertebrate species all contain the two conserved miR-148/152 binding sites. In six vertebrate species there is an additional conserved binding site (data not shown). It has not been shown previously that miR-148 and/or miR-152 can target *Mitf.* However, both miRNAs have been shown to be downregulated in melanoma cells [30]. Microarray analysis revealed that miR-148b is downregulated in early melanoma progression, although it was not confirmed using qRT-PCR [30]. Similarly, miR-152 was shown to be downregulated in a highly invasive melanoma cell line (Mel Im) [30]. Other studies have suggested that miR-148 and miR-152 can play a role as tumor suppressors. miR-148 expression has been shown to be downregulated in tissue samples from undifferentiated gastric cancer compared to normal tissue [49]. Similarly, hypermethylation of the miR-148, 124a3 and miR-152 genes was found in 34-86% of primary human breast cancer specimens [50].

The binding sites for microRNAs miR-124/506 are very well conserved in the *Mitf* 3′UTR sequence. Ten vertebrate species contain both miR-124/506 binding site; the *Loxodonta africana* sequence only contains 124/506B. It has not been shown previously that miR-124 and/or miR-506 can affect *Mitf* mRNA. However, miR-506 was shown to be upregulated in early melanoma progression and downregulated later in metastatic melanoma [30] and may therefore play an important role in this cell type. miR-124 is predominantly expressed in the brain, specifically in differentiating and mature neurons [51], [52], [53]. miR-124 is interesting considering its expression in the retina [54], as *Mitf* plays an important role in eye development. *Mitf* is expressed in the retina early in development and is later downregulated in this tissue [55], [56], [57]. The mechanisms that mediate this downregulation have not been described and may involve miR-124. However, our results show upregulation by miR-124 in melanoma cells, rather than downregulation. The possible role of miR-124 in regulating the expression of *Mitf* during eye development needs to be tested further. Although miRNAs are better known for downregulating target genes, there have been reports on effects through upregulation by miRNAs by binding to their promoter region [58], [59]. However, this is unlikely to play a role in regulating *Mitf* mRNA through effects on the 3′UTR since our reporter construct only contained 3′UTR sequences.

It has been shown that two thirds of genes targeted by miRNA have alternative 3′UTRs. The microRNAs miR-137 and miR-148 have many other potential target genes according to online prediction programs (Targetscan). In humans, miR-137 has 857 conserved targets, with a total of 949 conserved sites and 120 poorly conserved sites. miR-148 has 536 conserved targets, with a total of 581 conserved sites and 134 poorly conserved sites. Of these potential target genes, 66 are common targets of both miR-137 and miR-148. It is therefore unlikely that these miRNAs exclusively affect *Mitf* expression.

As miRNA binding sites are often located close to polyadenylation signals (PAS) [60], shortening of the 3′UTRs may lead to loss of miRNA binding sequences and thus loss of microRNA-mediated regulation. The mouse *Mitf* 3′UTR sequence contains twelve potential PAS sequences (two are AAUAAA, four AUUAAA, four AAGAAA and two UAUAAA, there is no AGUAAA sequence). Comparing these potential PAS sites in the 3′UTR sequences of *Mitf* from 11 vertebrate species revealed a well conserved PAS located at 2837-2842, adjacent to the miR-137 C binding site (Fig. 1A). Ten species contain a PAS at this location. The PAS located closest to the 3′end is conserved in all species and represents the signal for the longest mRNA version. Several other PAS's are well conserved whereas others are less conserved. miRNAs can have an impact on cancer progression in several ways, including loss of expression of a tumor-suppressor miRNA gene and over-activation of an oncogenic miRNA. Loss of miRNA binding sites in 3′UTR sequences of the target genes caused by mutations or translocations are also possible. Shortening of the 3′UTR sequence, and therefore loss of miRNA binding sites, can lead to oncogenic activation [61]. The conservation of several of the alternative polyadenylation signals found in the 3′UTR sequences of *Mitf* suggests their significance. Thus, *Mitf* might be able to evade miRNA-mediated regulation using the alternative polyadenylation sites, thus leading to a shorter transcript lacking the miRNA binding sequences. It was recently shown that *MITF* mRNAs with shorter 3′UTR sequences are more abundant in melanoma cell lines than transcripts with full-length 3′UTR sequences [44]. In this case the miR-340 is involved in regulating the shorter transcript and, interestingly, the RNA binding protein CRD-BP prevents binding of the miRNA. Thus, in the case of *MITF*, microRNAs do not act alone in mediating regulation through the 3′UTR sequences. Interestingly, the MITF protein has been shown to bind to the promoter of miR-148b that is shown there to have functional target site within the *Mitf* 3′UTR sequence [62]. This indicates a possible negative feedback loop where higher amounts of MITF would lead to more miR-148 expression which in turn adjusts MITF protein at desirable levels.

It is clear that miRNA function is diverse and its role in cancer and other disease is complex. Some miRNAs seem to have a tissue-specific function like miR-124 in neurons wheras other miRNAs are found more universally. *Mitf* functions in diverse cell types and may therefore be regulated by different miRNAs in the different tissues. In melanoma cells, *Mitf* is regulated by miR-137 and miR-148/152. As *MITF* is a lineage survival oncogene in melanoma [23] whose expression needs to be maintained at a certain level, it is likely that these miRNAs play an important role in regulating *MITF* mRNA levels in this cell type. Dysregulation of these microRNAs, or loss of the binding sites in the 3′UTR, might lead to increased expression of *Mitf*, thus leading to differentiation and cell-cycle arrest. As the miRNAs involved may have significant therapeutic potential, it will be important to characterize this regulation in more detail in further studies.

## Supporting Information

Table S1 P-values for data presented in Figures 2 and 3.
(0.02 MB PDF)
