# Supplementary material for: miR-148 Regulates Mitf in Melanoma Cells
Source: PLoS One. 2010 Jul 14;5(7):e11574. doi: 10.1371/journal.pone.0011574 (PMC2904378; doi:10.1371/journal.pone.0011574)
Supplement: Table S1 — P-values for data presented in Figures 2 and 3. (0.02 MB PDF) [file pone.0011574.s001.pdf]

**Figure 2A. P-values**<br>miR-182 0,1 pmol = 0.7937; 0,5pmol = 0.2103; 1 pmol = 0.6109.<br>miR-neg#1 0,1 pmol = 0.2123; 0,5pmol = 0.3279; 1 pmol = 0.4472.<br>miR-27 0,1 pmol = 0.1583; 0,5 pmol = 0.0190; 1 pmol = 0.2792.<br>miR-32 0,1 pmol = 0.4246; 0,5 pmol = 0.1268; 1 pmol = 0.1216.<br>miR-101 0,1 pmol = 0.1646; 0,5 pmol = 0.0282; 1 pmol = 0.0147.<br>miR-124 0,1 pmol = 0.5204; 0,5 pmol = 0.216; 1 pmol = 0.3813.<br>miR-137 0,1 pmol = 0.2495; 0,5 pmol = 0.1241; 1 pmol =0.4852.<br>miR-148 0,1 pmol = 0.0540; 0,5 pmol = 0.0037; 1 pmol = 0.0015.
**Figure 2B. P-values**<br>miR-182 0,1 pmol = 0.0047; 0,5 pmol = 0.0013 and 1 pmol = 0.0057.<br>miR-27 0,1 pmol = 0.0041; 0,5 pmol = 0.2825 and 1 pmol = 0.1265.<br>miR-32 0,1 pmol = 0,1293; 0,5 pmol = 0.0271; 1 pmol = 0.2190.<br>miR-101 0,1 pmol = 0,838; 0,5 pmol = 0.8558; 1 pmol = 0.3407.<br>miR-124 0,1 pmol = 0.0444; 0,5 pmol = 0.0001; 1 pmol = 0.0015.<br>miR-137 0,1 pmol = 0.0002; 0,5 pmol = < 0.0001;1 pmol = 0.0001.<br>miR-148 0,1 pmol = 0.8978; 0,5 pmol = 0.1053 and 1 pmol p-value= 0.0046.
**Figure 2C. P-values**<br>124+27 <0.0001; 124+32 = 0.0002; 124+101 <0.0001; 124+137 = 0.0063;<br>124+148 = 0.0001; 124+182 = 0.0227; 137+27 = 0.0007; 137+32 <0.0001;<br>137+101 = 0.0016; 137+148 = 0.0013; 137+182 = 0.0008; 148+27 = 0.0005;<br>148+32= 0.0252; 148+101 = 0.1101; 148+182 = 0.2513; 27+32 = 0.2761; 27+101 = 0.2468;<br>27+182 = 0,4290; 32+101 = 0.3511; 32+182 = 0.6395; 101+182 = 0.0400.
**Figure 3A. P-values**<br>Luciferase (without the Mitf-3´UTR sequence) + miR-148 = 0.3771.<br>Mitf-3´UTR-luciferase + miR-148 = 0.0140.<br>Mitf-3´UTR-luciferase + miR-neg#2 = 0.0829.<br>Mitf-3´UTR-luciferase + miR-182 = 0.6294.<br>Mitf-3´UTR-luciferase mut 148/152A + miR-148 = 0.0049.<br>Mitf-3´UTR-luciferase mut 148/152B + miR-148 = 0.3929.<br>Mitf-3´UTR-luciferase mut 148/152A+B + miR-148 = 0.4918.
**Figure 3B. P-values**<br>Luciferase (without the Mitf-3´UTR sequence) + miR-137 = 0.0002.<br>Mitf-3´UTR-luciferase + miR-137 = 0.0015.<br>Mitf-3´UTR-luciferase + miR-neg#2 = 0.1071.<br>Mitf-3´UTR-luciferase + miR-182 = 0.0064.<br>Mitf-3´UTR-luciferase mut 137 A + miR-137 <0.0001.<br>Mitf-3´UTR-luciferase mut 137 B + miR-137 <0.0001.<br>Mitf-3´UTR-luciferase mut 137 C + miR-137 = 0.0001.<br>Mitf-3´UTR-luciferase mut 137 D + miR-137 = 0.0001.<br>Mitf-3´UTR-luciferase mut 137 C+D + miR-137 <0.0001.
**Figure 3C. P-values**<br>Luciferase (without the Mitf-3´UTR sequence) + miR-124 = 0.0152.<br>Mitf-3´UTR-luciferase + miR-124 = 0.0086.<br>Mitf-3´UTR-luciferase + miR-neg#2 = 0.9666.<br>Mitf-3´UTR-luciferase + miR-182 = 0.0509.<br>Mitf-3´UTR-luciferase mut 124/506A + miR-124 = 0.1376.<br>Mitf-3´UTR-luciferase mut 124/506B + miR-124 = 0.0030.<br>Mitf-3´UTR-luciferase mut 124/506A+B + miR-124 = 0.1864.
